# The transnational mental health burden of Haiti’s alleged collapse: preliminary findings from The Haitian Well-Being Study

**DOI:** 10.3389/fpubh.2024.1415650

**Published:** 2024-09-27

**Authors:** Judite Blanc, Niara J. Carrenard, Evan Auguste, Sherryn Luma, Laura Francois, Tim Bigdeli, Girardin Jean-Louis, Lunthita Duthely

**Affiliations:** ^1^Department of Psychiatry and Behavioral Sciences, University of Miami Miller School of Medicine, Miami, FL, United States; ^2^Department of Psychology, University of Massachusetts, Boston, MA, United States; ^3^School of Nursing and Health Studies, University of Miami, Coral Gables, FL, United States; ^4^Public Health Academic Bldg (PHAB), SUNY Downstate Health Sciences University, Brooklyn, NY, United States; ^5^Department of Obstetrics, Gynecology, and Reproductive Sciences and Department of Public Health Sciences, University of Miami School of Medicine, Miami, FL, United States

**Keywords:** Haitian traumas, violence, disaster management, Haitian Well-Being, generational healing, mental health

## Abstract

**Background:**

Transnational drug trafficking, political unrest, gang violence, and paramilitarism, which are pervasive in Haiti, have resulted in a mental health crisis for the broader Haitian community. This study explores the mental well-being of Haitians in Haiti and the United States by identifying barriers and facilitators to mental health through the lived experiences of men and women.

**Method:**

Four Focus group discussions conducted in April and November 2023 engaged 28 participants (20 women and eight men) aged between 23 and 60 years from locations in Haiti (Port-au-Prince, Cite Soleil, Cayes, Cap-Haitien, Saint-Marc) and the United States. Discussions revolved around the definition of mental health, stressors, coping mechanisms, risk and protective factors, and barriers to mental health care.

**Results:**

Six principal themes emerged: 1- *Chronic Traumatic Stress*: continued violence, political instability, unemployment, lack of social support, adverse childhood experiences, family separation, and forced displacement were significant sources of stress. 2- *Increased Health Burden*: Participants reported experiencing chronic physical and psychological symptoms [i.e., hypertension, anxiety, depression, sleep issues, substance abuse, suicidal ideations, characteristics of post-traumatic stress disorder (PTSD)], which were attributed to Haiti’s social, political, and infrastructure collapse. 3- *Risk Factors*: limited access to mental health services, pervasive hopelessness, scarcity of opportunities, and stigma were identified as significant risks. 4- *Future Uncertainty*: widespread concerns regarding the future predominated. 5- *Multigenerational Concerns*: Significant anxiety concerning the mental health and development of children, as well as the functionality of mental health practitioners, was noted. 6- *Coping and Protective Factors*: Effective coping strategies include mental stimulation, peer support, managing digital consumption, engaging in leisurely activities, such as listening to music, and faith/spirituality.

**Conclusion:**

The study’s findings underscore the sociopolitical and economic crisis in Haiti, which has resulted in violence and a dismantle of political, educational, financial, and health infrastructures. These factors were identified as the primary source of chronic distress, contributing to widespread mental health issues, adverse physical symptoms, and disruption in daily life. The implications for practice, healing, research & policy are discussed.

## Introduction

On March 4, 2024, subsequent to the seizure of major airports across Haiti and a widespread prison break orchestrated by the paramilitary coalition known as Federation G9 Family and Allies, the Haitian government declared a state of emergency (1). This event unfolds amidst a backdrop of persistent unrest following prolonged anti-corruption protests against the Moïse regime, marked by interruptions such as Moïse’s assassination in 2021, the rise and resignation of unelected Prime Minister Ariel Henry, and the simultaneous control of the capital by disparate gang and paramilitary factions (2, 3). Both residents on the island and members of the Haitian diaspora increasingly recognize the direct linkages between transnational drug trafficking, international political interests, and the escalating violence on the ground (4–6). Consequently, the Haitian populace finds itself ensnared in a cycle of intermittent violence, with a majority of firearms sourced from the United States (6), while the United Nations and Core Group officials deliberate over the political fate of the nation. Moreover, looming is the potential deployment (at the time of this writing) of a US-trained Kenyan police force notorious for extrajudicial killings of distinct ethnic groups (7). As highlighted by historian Jean Casimir, this predicament reflects Haiti’s enduring legacy of never truly exercising self-governance in accordance with the will of its people (8).

Amidst this ongoing political and humanitarian turmoil, Haiti faces a severe mental health crisis. While in the past, a diverse array of practitioners both on the island and within the diaspora attended to mental health needs (9), the protracted period of violence has resulted in the collapse of many public systems and the exodus of numerous Haitian professionals. Nevertheless, in alignment with decolonial approaches to mental health, the collective voices of the Haitian populace remain pivotal in formulating, advocating for, and disseminating mental health and political solutions.

This study is grounded in theories of multigenerational trauma responses, as well as social determinants of mental health (10, 11). Specifically, we considered how the structural legacies of colonialism and enslavement shape the psychological conditions of current Haitians across distinct generations. As throughout much of the global south, Haiti’s infrastructure reflects legacies of colonialism and exploitation (2, 12–15). Such conditions have shaped the disparate exposure to traumatic events for Haitian people (2). Multigenerational trauma often considers intergenerational pathways of risk and thus may manifest as a cyclical mechanism deeply entrenched in Haitian history and sociopolitical climates, resulting in adverse effects on individuals residing in Haiti and abroad (16). Historical trauma, defined as “cumulative emotional and psychological wounding over the lifespan and across generations, emanating from massive group trauma experiences” (17), is particularly salient when examining mental health, as evidenced by recurrent themes of physical, behavioral, cognitive, and psychiatric manifestations among Haitian families and their descendants, serving as a conduit for intergenerational transmission of stress (18).

This complex web of historical, social, and psychological factors underscores the urgent need for comprehensive and culturally sensitive approaches to addressing large-scale mental health challenges in Haiti. While external assistance and interventions can play a supportive role, any sustainable solution must center on empowering local communities and incorporating indigenous healing practices. Collaboration between local stakeholders, international organizations, and researchers is essential in developing and implementing evidence-based interventions that are tailored to the unique sociocultural context of Haiti.

The current project is among the first qualitative studies focused on the psychological distress and needs of Haitian people during the current crisis. Specifically, focus groups of Haitian people living through the current crisis shared their (1) understanding of mental health, (2) the factors leading to distress, and (3) coping mechanisms.

## Method

### Study design and participants

Data collection was approved by the Institutional Review Board of the University of Miami Miller School of Medicine. Data were de-identified and anonymized. Focus group participants were part of the larger Haitian Well-Being Study Phase 1 (IRB#: 20211103; November 2022), a pilot project aimed mainly at identifying the psychosocial, neurological, genetic, sleep, physical health, socio-economic (SES), spiritual, and health behaviors of a diverse sample of Haitians living in Haiti and the United States.

Participants were made aware of the study through a flier shared via email with community-based organizations (CBOs), health forums, study social media, and text messages to individuals with whom they had pre-existing relationships. Inclusion criteria were individuals who were (a) 18 years of age or older, (b) spoke English or Haitian Kreyol, and (c) resided in Haiti or identified as people of Haitian descent living in the U.S. Exclusion criteria were (a) individuals who had cognitive impairment that would preclude completion of the surveys or reduced capacity to understand risks and benefits of the study, (b) non-English or non-Haitian Kreyol speakers, and (c) did not identify as a person of Haitian descent.

**Figure 1:**
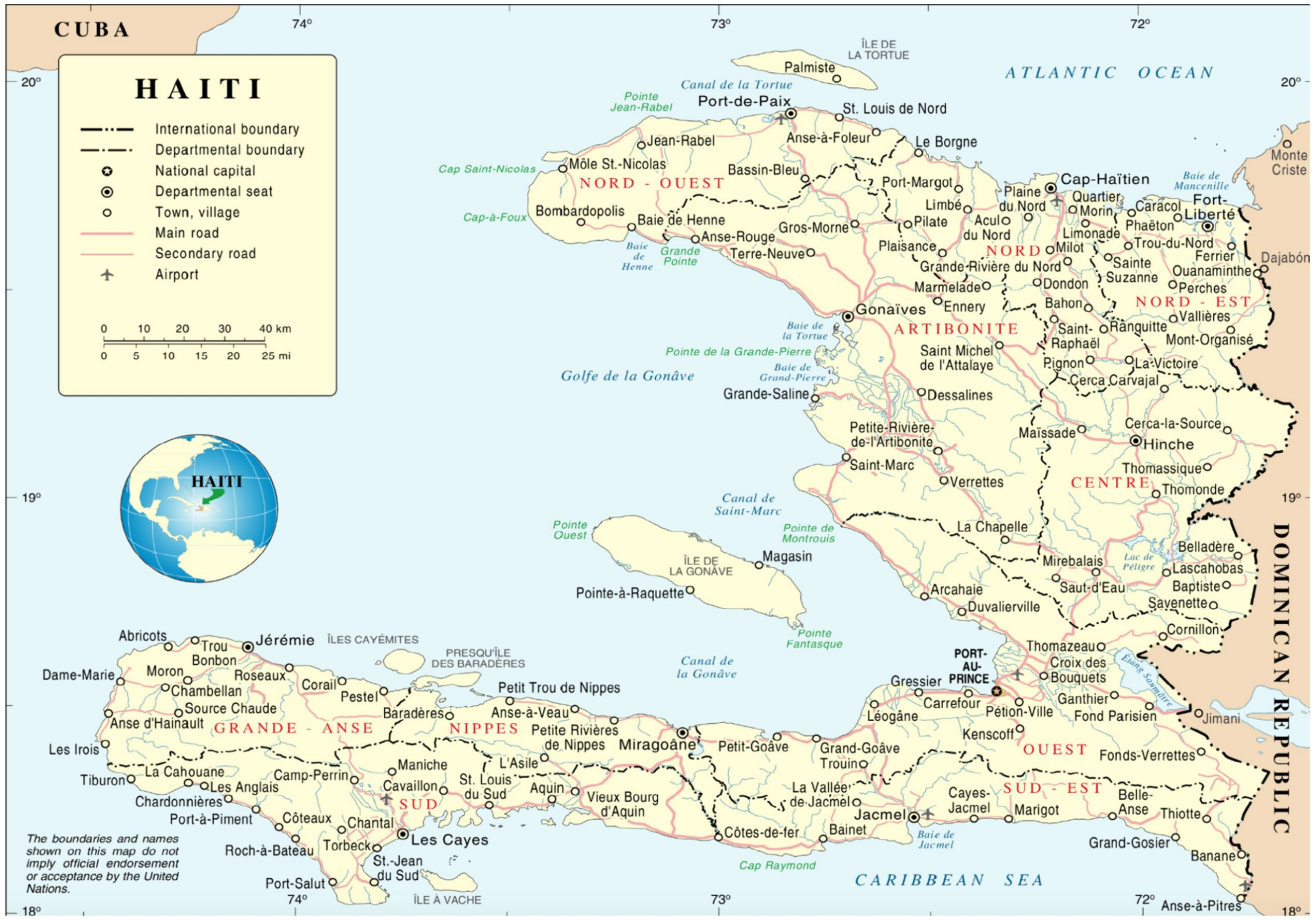
Haiti’s Departments and International Boundaries based on the United Nations (UN) Map of Haiti.

A total of 28 participants (20 women and eight men) between the ages of 23 and 60 years old (*M* = 29.5, SD = 9.8), were included in the study. The participants were situated in various cities, with the majority residing in Cite-Soleil, one of the most impoverished and dangerous slums (see Figure 1) in Port-au-Prince (western department), Haiti (39.3%), and others residing in Les Cayes, Haiti (southern department) (14.3%), Port-au-Prince, the capital of Haiti (7.1%) (western department), Cap-Haitien, Haiti (second most populous city in Haiti located in the northern department) (3.6%), Saint-Marc, Haiti (western and Artibonite regions) (3.6%). The United States, home to the highest number of Haitian citizens outside of Haiti, is also the main site of the Haitian Well-Being Study. Several U.S.-based participants provided their city but not their state, with participants calling from Miami, Florida U.S. (3.6%), New York, U.S. (3.6%), and Spring Valley, U.S. (3.6%). In addition, 4 participants solely listed their country of residence (7.1%, Haiti; 7.1%, United States), and 1 participant did not disclose their location.

### Data collection

Focus groups were conducted using a semi-structured interview guide developed to elicit participants’ perspectives on mental health, sources of distress, coping mechanisms in the context of the ongoing crisis. The interview guide was designed based on a review of the literature on mental health in conflict-affected settings and was informed by consultations with Haitian mental health professionals and community leaders.

Focus groups were conducted in either Haitian Kreyol or English, depending on the participants’ preference. Each focus group session lasted approximately 90 min and was audio-recorded with participants’ consent. The recordings were transcribed verbatim and, where necessary, translated into English for analysis. To ensure the accuracy of the translations, bilingual members of the research team reviewed the translated transcripts.

### Procedures: focus group

Participants who expressed interest in the study received a link to a website to consent to participate. The online survey was administered through RedCap, a secure, widely used data collection platform. Data collection involved the use of focus groups conducted remotely. Focus groups were conducted between March and November 2023 in Haitian Kreyol and online via an encrypted Zoom meeting. Focus groups lasted approximately 60–90 min and were facilitated by the PI (a Haitian-born clinical psychologist). A research team member (a Haitian-born medical doctor) was present as well. With participant consent, focus groups were video and audio recorded. Focus groups, as an interview methodology, were chosen as they provided the ability to capture diverse perspectives and to explore complex social phenomena through group interaction and discussion (19, 20).

At the beginning of each focus group, guidelines co-created by the PI and research team member, were discussed with participants, prior to the start of the focus groups. Focus groups participants were ensured about confidentiality and focus group rules. Participants demonstrated their understanding with a raise of hands. Table 1 provides a breakdown of the domain and corresponding sample interview questions. During the focus group discussions, participants had the opportunity to discuss overall mental health and well-being, catalog past and present stressors, explore risk and protective factors in mental health, and identify primary barriers to accessing mental health care. The sessions were structured to encourage an open and guided dialogue, allowing for a comprehensive exploration of each participant’s experiences and insights.

**Table 1:** Focus group guide.

Domain	Questions
Determining overall mental health and well-being	When you hear the term “mental health” what comes to mind?
	How would you describe your mental health?
	How do you take care of your mental health?
	How do you help your loved ones take care of their mental health?
Identifying past and current stressors	How do you define ‘stress’?
	What are the most common stressful situations you have faced?
	What are the most common mental health concerns people have faced and/or are currently facing in the Haitian community?
	What are the most stressful things for you, right now? (Optional)
	What stressors do you think members of your community are experiencing?
	What do you think are the reasons you and/or people in your community may be dealing with these stressors?
Understanding risk and protective factors for stress and well-being	Why do you think Haitians do not seek help for mental health issues?
	How do you cope with the latest stressful events affecting the Haitian communities?
	Have you been taught how to cope with stress and mental health issues?
Health equity barriers	Where do you access mental health services and resources? (If not, where would you go for care?)
	Have you sought out support for mental health concerns? Why or why not?
	Are you and/or members of your community able to seek support?
	What barriers have you faced when seeking support?
	What barriers do members in your community face when seeking support?

### Data analysis

All focus group transcripts were first de-identified by the PI, and a research team member reviewed the transcript line by line and edited the text as necessary to ensure the accuracy of the transcription. Next, two research team members fluent in Haitian Kreyol translated the transcripts into English. The transcripts were then coded thematically using methods outlined by Braun and Clarke (21). The Braun and Clarke (21) method for thematic analysis is widely used for qualitative data coding. The analysis is conducted in six phases:

1. Familiarizing yourself with the data;
2. Generating initial codes;
3. Sorting the codes into themes;
4. Reviewing and refining themes;
5. Defining and naming themes;
6. Conducting the final analysis and producing the report.

In addition to the steps listed above, research team members began the analytics process, and interrater reliability was determined. To communicate the results, the PI used a narrative approach.

## Results

**Figure 2:**
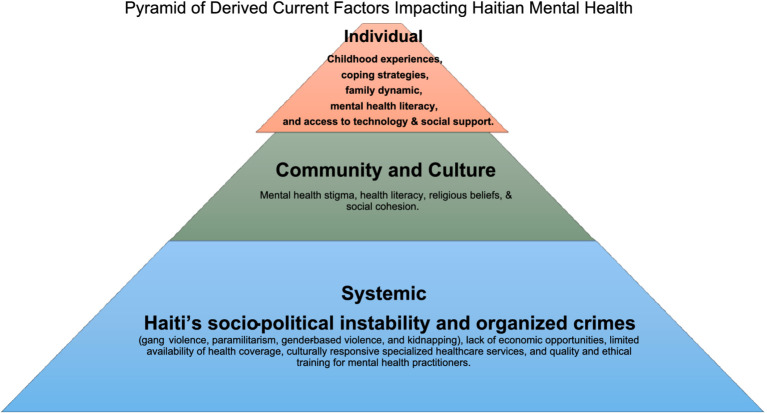
Pyramid of derived current factors impacting Haitain mental health.

Qualitative analysis revealed six key themes relevant to the mental health experiences of Haitian people, each encompassing a nuanced array of 14 sub-themes: (1) increased health burdens, (2) mental health risk factors, (3) chronic traumatic stress, (4) future uncertainty (5) multigenerational concerns, and (6) healthy coping and protective factors The thorough analysis identified a total of 346 references to themes and sub-themes (Figure 2 and Table 2) within the qualitative data. To protect participant identities, participants were assigned pseudonyms.

**Table 2:** Frequency of Themes and Sub-themes.

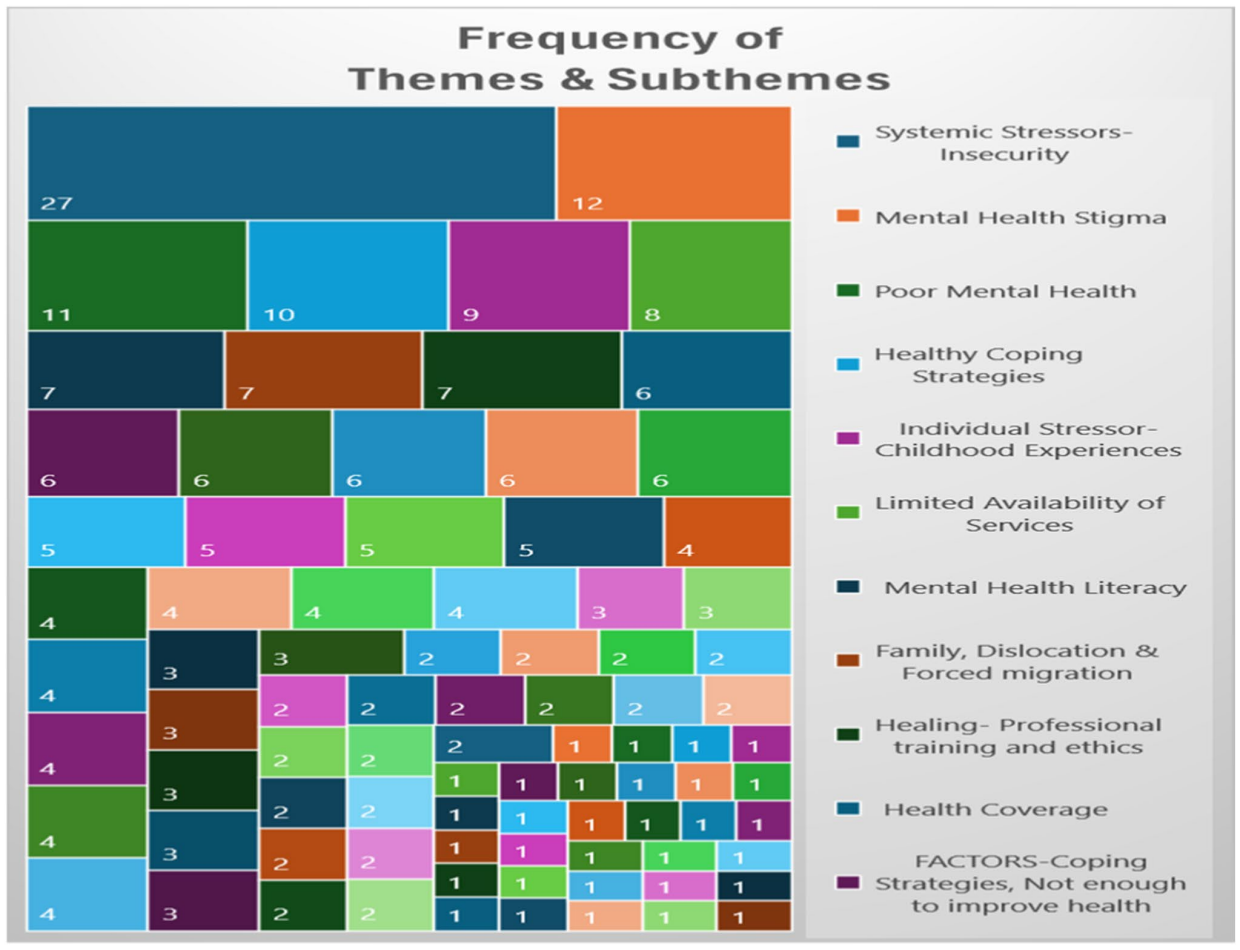

Table 2 itemizes the frequency with which various themes and subthemes, represented by specific colors and headings, emerged during focus group sessions conducted with participants from both the Haiti and United States cohorts. The tree map visually illustrates the prevalence of these mental health-related themes. The most prominent theme, “Systemic Stressors—Insecurity,” depicted in dark blue, appears 27 times, underscoring its significant impact. Other notable themes include “Mental Health Stigma” (12 mentions), “Poor Mental Health” (11 mentions), and “Healthy Coping Strategies” (10 mentions). Themes such as “Limited Availability of Services,” “Mental Health Literacy,” and “Family, Dislocation & Forced Migration” also appear with moderate frequency, while smaller subthemes are less common. This chart effectively highlights the key issues affecting mental health within the study, with systemic insecurity emerging as the most critical concern.

### Chronic traumatic stress stemming from organized crimes—*“Ou leve e ou tande bri bal* “*➔ “You wake up and hear the sound of bullets”*

Haiti’s history is marred by race-based enslavement, colonial looting, and gang-related, political, and sexual violence, compounded by unemployment and deficits in social infrastructure. It should be noted that some have criticized the use of the term “gang” to describe the violent actors throughout Haiti, as it has been used to drive racist narratives (22). Within this project, the term gang is used to reflect how participants described the violent actors, and not to diminish the connections between local violent actors and transnational drug and colonial interests. Amidst the flickering candlelight of the virtual focus group room, the atmosphere heavy with the weight of shared experiences, a somber truth emerged. Traumatic childhood experiences and forced displacement and migration further deepen the nation’s wounds. The prevalence of current violence/insecurity, mentioned 27 times during four focus group sessions, only serves to exacerbate the crisis.

#### Ongoing political and gang-related violence—“Strès se pa rapò jan peyi a vin evolye a pou m’ dòmi nan nwit li fè m’ leve a 11 du swa pou m’ kouri sa” ➔ “Stress is related to how the country has evolved. When I sleep at night, I often wake up at 11:00 PM to run”

Participants gave harrowing narratives speaking to the rampant gun violence and sexual assault. Marline, a young nursing student from Cite Soleil (Haiti) shared:

*My stress comes from how the country is evolving. When I sleep at night, it makes me wake up at 11:00 pm to run, which is a big stress for me because I’m not stable. In a moment when it gets too late when I’m not here or outside, it will be midnight, and I’m running from bandits.*

Rose (from Cite Soleil) described conditions as so dangerous that you cannot go outside.

*I do not have stable mental health compared to the situation in the country, you cannot sleep well at home. Sometimes it’s shooting. Sometimes you cannot go outside for several days. You cannot go to school. All of it seems to mess with our heads, we are not comfortable.*

Jessika (20, Cite Soleil) explained:

*The way the country is developing, imagine every day you wake up, if today you wake up and hear the sound of bullets, you cannot go out or go to school. And plus, the price of everything you buy is going up. Things are a bit not too good in Haiti, that’s what makes my mental health a bit bad.*

Natalie (23, Cite Soleil) describes the shared stress, stating:

*The stress comes from the situation in the country, and that’s also the source of my stress, because the way things have become is not safe, there is not a place to relax and have some fun. If you would like to take a breath of fresh air, if you would like to think about some other things you feel your mind is troubled. A person you just saw will die without them being sick. So all of this, they really stress us out, all of the stress affects our mental health.*

Natalie (23, Cite Soleil) shared “outside by my house, the other day I heard that a man was beheaded because he raped a 4-year-old child.” She continued with another incident of sexual assault that occurred to a friend.

*She kept crying. Her clothes and hair were messed up. She was scared to speak… she explained that a young man she used to date, whom she does not date anymore, went and told a bunch of other men that, and they took her, and they went somewhere else with her and raped her. After that, they dropped her off at her house.*

#### Forced migration and transnational mental repercussion—“Koman sa pral ye? Tout moun ap kite kounya” ➔ “How will it end up being? Everyone is leaving now”

Some related references extended to the Haitian diaspora in the US, including comments about how U.S. migratory policy impacts the Haitian expat community regarding immigration and cultural and political outcomes.

Stevenson (40, from Spring Valley, United States) lamented —.

*When you look at the community, for example, the community is present here, but mentally, the Haitian community is in Haiti. So this is having an effect, everything that is happening in Haiti has a repercussion on the mental health of the Haitian community here, especially in Spring Valley, where I am fortunate to live; When safety is an issue for them (in Haiti), it has an effect here.”*

Forced migration and displacement emerged as a significant concern during the focus groups, with participants grappling with the decision to leave Haiti for better opportunities amid ongoing adversities. Francesca (23, Cite Soleil) illuminated the impact of forced migration on essential services, lamenting the departure of healthcare professionals due to safety concerns. Despite the hardships, many understood the reasons behind the exodus, acknowledging the precariousness of the situation in Haiti.

*At the moment, people are still being shot, people are still dying because of the insecurity problem we live in. Imagine if I had a hospital near my house that was closed because all the doctors, hospital owners, and nurses were all left in Biden.*^1^ *We would also like to go, but we cannot go because we do not have anyone. When we look closely, we understand why they go because the country is not safe.*

Francesca lamented,

“People cannot eat, but that does not stop bandits from entering the hospital to take them. I can understand this part that makes them go, but I also do not understand why they leave us like that.”

Yvette (32, Les Cayes) expresses uncertainty about her future and also what would happen if someone did leave Haiti. She states:

*I personally cannot say that my mental health is good because I think a lot. There are many things that I would like to achieve, but unfortunately, I cannot yet because you are thinking in a month, in 2 months, how it will be. I can leave through Biden, but 2 years later, after Biden, what will happen? Nobody knows.*

Stevenson describes the essence of forced migration and displacement.

*You see your country falling apart, which means I do not think there are two people who would forcefully choose to leave their country to go somewhere else if they did not have to. I also think that it is very stressful for many people to see that you are obliged to leave your home and not stay, so you are not comfortable.*

#### Kidnapping—“Si paran an pa gen ranson an, se lanmò” ➔ “If the parent does not have the ransom, it is death”

Kidnapping emerged as a distressing reality for many participants, as Rose vividly described its prevalence in Haiti. She painted a grim picture of the situation, highlighting how kidnappings occur frequently and indiscriminately, striking fear into the hearts of the population. Rose’s account underscored the pervasive sense of insecurity and vulnerability faced by Haitians on a daily basis.

*It is something that happens every hour. In Haiti, all the people are affected by kidnapping, you hear all the time that they are taking people, you hear all the time that there are kidnappers, and you hear all the time that there is a child whose parents do not see them anymore, they have kidnapped them. And when that happens, the first thing they do to them is rape them. After that, they demand ransom. If the parent does not have the ransom, it is death.*

**Figure 3:**
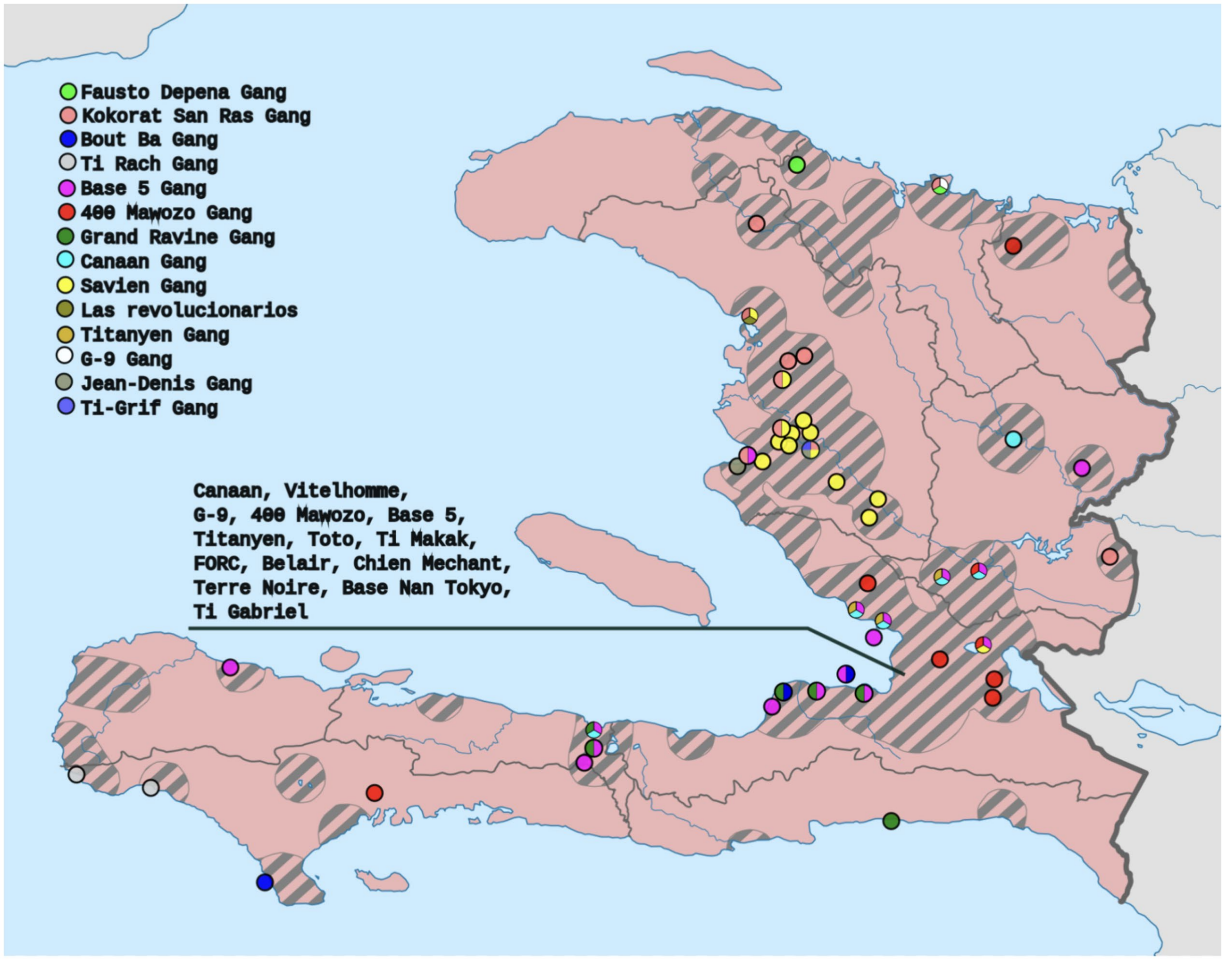
Map by Clyde H. Mapping derived from the data of Armed Conflict Location and Event Data Project.

Further, Marie (41, a woman from Miami while traveling back and forth to Haiti) depicts:

*The two most stressful things I can see for the community right now is the fact that kidnapping is very prevalent, which means that now it can happen to a person of any class when before it was said that it would be in the high-class people with money* etc. *Now anyone can be kidnapped in the country; I think this has become a huge stress factor for many people because it is just a violation of who you are* (see Figure 3).

### Increased health burdens— *“gen jèn gason de trantan, karantan k ap fè de kriz kadyak” ➔ “there are young men in their thirties and forties who are having heart attacks”*

Participants spoke of a silent epidemic sweeping through Haiti’s communities, leaving in its wake a trail of physical and emotional wreckage. As the discussion unfolded, tales of quiet suffering spilled forth, each narrative a testament to the toll exacted by Haiti’s worsening situation. With voices trembling with emotion, participants painted a vivid picture of the relentless onslaught of stress, anxiety, and despair. They spoke of sleepless nights haunted by fear, of hearts pounding in rhythm with the chaos outside their windows. Through tear-stained confessions, they revealed the hidden scars of collective trauma etched upon their psyches. Anxiety clenched like a vice around their hearts, while depression cast a shadow over their every waking moment. Substance misuse offered fleeting respite from the pain, but its grip only tightened with each passing day. In the hushed confines of the virtual focus group space, the collective weight of their shared burden hung heavy in the air.

#### Cardiovascular health outcomes—“Mwen se yon moun ki pa t janm gen pwoblèm tansyon avan”➔ “I am someone who has never had problems with blood pressure before”

One participant, Marie, vividly described a drastic increase in her blood pressure:

In February, two years ago, I went to the doctor’s office. It was 17*10. If one knows blood pressure, that is extraordinarily high. Now I am on blood pressure medicine when before, I was someone who was always healthy and had no problems with blood pressure, cholesterol, or anything. I think that this is a stress factor for many people in the community. There are too many young men in their thirties and forties who are having heart attacks.

Another participant, Nadège, (29, from Les Cayes), stated:

*I have developed something in myself that makes my heart beat fast, I get palpitations, and sometimes I feel that I am not the same person anymore because of stress and insecurity, so what pushes me to make a firm decision, I say that I cannot be productive if I leave.*

### Depression and suicide ideation—“Akoz de strès lan, m konn menm bliye pou kisa m ap kriye” ➔ “Because of my stress, I often forget why I am crying”

Natalie (from the Cite Soleil commune in Port-au-Prince, Haiti) shares:

*I get so stressed that I cry. I cry for so long because of the stress that I even forget why I am crying, and as a result, the stress makes me unable to eat, I’m hungry, but I cannot even eat food while I’m hungry, it usually makes me think of many things, and sometimes, I feel like I could kill myself. It is because I’m a Christian that makes me not kill myself because every time the thought of killing myself comes to mind, I say if I kill myself, I will not be saved. That’s why I have not killed myself already. Oftentimes I get to the point where it’s more than depression.*

Natalie (23, Cite Soilel) disclosed the following:

*I do not have good mental health. This is because sometimes I see how my parents live, I can see how things should be going for me, but they do not, and that makes me upset, and sometimes I cry. Well, as far as my mental health, I feel like I do not have good mental health. So I feel… I do not know if I can say it’s an illness… it’s a mental illness, but I do not feel I have good mental health.*

Similarly, Micheline (31, from Les Cayes, Haiti) stated, “My mental health is not so good. I think a lot.” Another Les Cayes participant, Sarah, described:

*My mental health is − 0. I’m in a situation where, with all the obstacles that I face, I do not react. I do not know what it’s called, how would I, what name would I give it, does it have a name in mental health, so even if, for example, someone were to tell me that someone is dead, even if they were close to me, I grew up with them it does not shake me. I get up, I do not react to it.*

### Health risk factors—*“Ayiti gen tout fasèt nèt ki fè yon ayisyen oblije ap fè gwo trajè sa”➔ “Haiti has all the ways that makes a Haitian have to go through this long journey”*

As the stories of struggle unfolded, a common thread began to weave its way through the shared experiences. Across all focus groups, a stark realization emerged: the landscape of mental health in Haiti is marked by scarcity, neglect, and stigma. Participants spoke of the Herculean task of seeking mental health support in a country plagued by scarcity. With voices tinged with frustration and resignation, participants from Haiti and the US lamented the dearth of qualified practitioners, the absence of healthcare coverage, and culturally responsive services. For many, the healing journey was fraught with obstacles, with access to care hindered by geographical distance, financial constraints, and a lack of infrastructure. Against this backdrop of scarcity, hopelessness loomed large, casting a shadow over the collective psyche of the nation. Participants spoke of dreams deferred, opportunities squandered, and futures dimmed by the relentless grind of adversity. In a country where the pursuit of mental well-being was often seen as a luxury afforded only to the privileged few, hope was a scarce commodity.

#### Barriers to accessing care— “Mwen poukont mwen e mwen vle èd” ➔ “I am by myself and I want help”

“I do not think there are enough psychologists left in the country at the moment to go see even though we are going through a very, very delicate situation,” stated Francesca.

Yvette, a participant from Les Cayes, shared:

*So there is not really a psychologist who remains open in Haiti, who has an office, who accepts people for sessions, who hears you. There is not really an office like this except in a hospital that has a center, I do not really remember the names, but where they receive people who have HIV, people who have had a positive AIDS test,* etc.*, you will find a place to do a screening, there is a psychologist there or in places that deal with violence against women, violence against children, so it is always in a center or an institution where there are these types of people.*

Joceline (53, from New York, United States) expressed her frustration over the lack of mental health professionals and effective coping mechanisms for stress in Haiti. She states:

*I have never seen people in my life find a good way to deal with stress. For example, my father is someone who is obsessed with sex, and my mother is someone who is obsessed with Christianity, she’s used to church, but none of these ways have ever done anything, their lives are still not good. My father died young, at 40 years old, and my mother’s life is over.*

Junior (27, Haiti), a participant in the men’s group, shared his personal experience of lacking access to mental health care despite feeling the need for it at times. He discloses:

*I personally did not have access to mental health care, and even though at a certain moment I felt I needed it, I did not have access to it. This is not to say that care is not available, services are not available, and they are not readily accessible because they must be available. There are psychologists, there are therapists, but are they accessible to a large number of people? I did not have anyone to tell me here was a counselor that I can talk to, here is somewhere could go if I have a problem if I did not feel well mentally, I could not find that. So maybe it’s available to some people, but I do not think it’s democratized, that’s what I’m saying. I’m also alone, and I want help. I’m the one doing the research. I’m the one looking for help. I’m the one looking on YouTube and others.*

Marline shared a similar viewpoint. She said, “…since it is Haiti, I must say that the psychologist is very rare, so that people would come to him to treat this disease, there would have to be a majority of people who are stable to do this, because it is minimal, it’s very minimal due to lack of money I cannot go to the next place to check a psychologist who would do this to help me,” indicating that not only is there a lack of mental health professionals available, there is also a financial barrier. As such, there is a need for democratization of help-seeking for mental health issues in Haiti.

#### Mental health stigma– “Si ou gen pwoblèm, ou fou” ➔ “If you have a problem, you are crazy”

For example, Sarah (31, Les Cayes) explained:

*It is true that the need is there, but there is not a concerned body that is in charge of them. But also, do not you think there is another factor that we should work on because you will not find a lot of people with a problem who will sit down and talk to a psychologist. So, because of us, in our society, when someone goes to see a psychologist, they say you are crazy, so I think there is work to be done with the people so that they can really know what the role of a psychologist is.*

*Getting mental health care has never been easy. People have always said that if you have problems, you are crazy. It’s because you are not are not strong enough. You are a person who is too nervous, you have too many problems.*

Joceline, New York.

#### Insecurity— “Ayiti gen tout fasèt nèt ki fè yon ayisyen oblije ap fè gwo trajè sa” ➔ “Haiti has all the ways that makes a Haitian have to go through this long journey”

Amidst the narratives of struggle and resilience, a chilling tale of desperation emerged, painting a vivid picture of the dire realities faced by many in Haiti. Stevenson, described the extreme measures taken by Haitians to escape hunger and insecurity. He says:

*When you hear a Haitian explaining the last wave before the Biden program, he tells you that he has traveled 11, 12, 13 times across the country on foot. So yeah, that shows you the level of degradation Haiti has reached security-wise. All of these facets are what make Haitians have to make this great journey in order to save themselves.*

Nadège (29, Les Cayes) explained:

*I myself can say that it is a problem of insecurity. The worst thing is that those in a position to solve the problem have no will to bring hope for the future.*

Further, there is an intergenerational concern for how future generations will be raised in these conditions. These narratives highlight the urgent need for efforts to address insecurity in Haiti and restore hope for the future.

### Multigenerational concerns— *“Enkyetid lan plis pou pitit yo ke pou tèt yo”* ➔ “the anxiety is more for their kids than for themselves”

The impact of Haiti’s tumultuous state extends beyond adults to affect its children profoundly. During discussions about addressing the current conditions, participants voiced deep apprehension for the well-being and prospects of the younger generation. Nadège voiced a pressing concern about the dire circumstances faced by Haitian children, emphasizing their vulnerability surpassing that of adults. She earnestly inquired about the existence of mental health initiatives tailored specifically for children, highlighting the urgency of prioritizing interventions to address their unique needs. Nadège’s poignant words underscored the collective anguish felt by many Haitians witnessing the plight of their children amidst the ongoing challenges gripping the nation.

*Children are living in situations of not only insecurity and problems of natural disasters, problems of uncertainty for tomorrow, but children are living it worse than adults… Will there be a possibility for your program to be able to bring an in-depth solution for mental health, children, youth, and the older adult in Haiti?*

Sharing similar sentiments, she noted that many parents care more for their children’s well-being than their own and that the children need psychological support. She shared:

*We, as parents, see how difficult it is for us. If we think about the children who are living this situation in a more complicated way, it is important to understand all the people who have children that you see and everything that they say… they are more concerned for their children than for themselves because it will be more difficult for the child to live and go through this moment, especially tomorrow, which is uncertain.*

Rose stated.

*…And for the problem of insecurity to be solved and for the expensive life to go down. Parents cannot send their children to school because there is no work. Life is hard. There are many times they cannot send their children to school. There are many parents who have to sit at home, and they cannot go out.*

### Future uncertainty— *“tout moun renmen viv nan peyi yo men eske kondisyon yo reyini?”➔ “everyone likes to live in their country, but have the conditions been met?”*

A prevailing sentiment among participants was uncertainty and apprehension regarding their future. Sarah, hailing from the Cap-Haïtien and Les Cayes focus group, articulated this sentiment poignantly. She expressed the internal struggle faced by many Haitians: the dilemma of whether to remain in their homeland amidst the turmoil or seek refuge elsewhere. Sarah’s words encapsulated the profound existential angst and lack of clarity that pervades the minds of many Haitians as they grapple with the uncertainty of what lies ahead in their strife-ridden nation.

*You are stressed in a country, so you do not know what future you have in a country, you have children, you have a husband, you do not know, am I supposed to stay here…but is going away the solution?*

Similarly, another participant, Jean, a 27-year-old medical resident (Saint-Marc), reported:

*One of my biggest worries is my future. I am currently in Saint-Marc in my social service. I am from Lyankou commune Vèret, where I have not gone home since 2020. Bandits have invaded the area.*

Francesca (from Cite Soleil, Haiti) states:

*Imagine an area invaded by bandits, these people are just running, they do not even know where they are going. They arrive and find another group of people sitting, and they sit down too. Even though they are hungry, they sit on the street; they have no money. It’s just like… even if they have their phones in their hands, they listen to music, they do this, they do that, they try to talk among one another in the same situation. They will always have in their mind that they will not be able to return to where they were before, and there is nowhere else to go.*

### Healthy coping and protective factors— *“Nou konnen nou ka konte sou lòt”➔ “we know we can count on one another”*

Yet, even in the face of such adversity, Haitian resilience persists, compelling us to take action to break the cycle of suffering. Amidst the turbulence of Haiti’s present circumstances, Haitian individuals have leaned on various coping mechanisms and protective factors to navigate the stress and uncertainty.

#### Familial/peer support and prayer—“Mwen priye anpil pou Bondye banm kouraj”➔ “I pray a lot for God to give me courage”

Ricardo (39, Saint Marc) described how important having family support was. He said:

*Having family support, especially at this time, is important. Find a friend you can count on. Solidarity is one of the protective factors. And what do we do when there are signs of distress? For example, believers can say that it is prayer. Some people may say it’s meditation, and some may say it’s music.*

Marline also noted prayer as a significant protective factor. She said, “To face this situation, I pray a lot for God to give me courage after I do a little music. Even though it will not do anything serious, it is the prayer that keeps me.” Similarly, another participant, Reynold (24, Haiti), described how spiritual practices serve as protection against the things he is currently experiencing. He states:

*We know that we can count on others, and it is also a protective factor and our belief, and sometimes it can even be a hope that allows us to understand what is happening because it will not stay like this, they will fall like rain, even the rain has a cause and this allows me to move forward with these situations. So it’s family, understanding, and of course, spiritual beliefs, spiritual practices, and prayer that can all be useful for me as a factor of protection.*

#### Creative and mind–body practices— “Dwòg pa m se mizik”➔ “My drug is music”

Another coping strategy mentioned by participants was the use of music or an enjoyable activity to cope. Stevenson explained:

*My drug is music. Well, I listen to a lot of music, and I realize that music helps me stay away from many things. A lot of things that I could have thought of or that could have been a stressful element for me, but music helps me to take a different turn since I’m in music, and I know practically nothing. I like sports a lot, I exercise from time to time, I always look for 30–45 min to exercise, and politics is also my passion… so I try to keep a balance in this way.*

Rose (21, Cite Soleil) poignantly expressed a sense of hopelessness, noting the lack of options and the pervasive insecurity in Haiti.

*For me, it (music) is the only means we have to fight stress in Haiti because there are problems with insecurity; you cannot go out, you have no place to go, and you cannot do anything.*

#### Unplugging— “Pa ale sou Facebook souvan” ➔ “Do not go on Facebook often”

“Unplugging,” defined as conscious and deliberate abstaining from using technology devices and platforms, which results from perceived overuse, is a strategy used to improve well-being (23). Managing digital consumption, or unplugging, is also a strategy identified as a protective factor by the focus group participants. Shielding oneself from stress-inducing information to maintain one’s mental well-being was a coping mechanism. Jean (27, Haiti) explained:

*If you want to protect yourself from these situations, you may just not use or go on Facebook often, and there are certain WhatsApp groups you can avoid. There are people who like to post about certain events, so you can always block these people from your contacts. This is the protective factor that I use. Whenever I do not want to be aware of what is happening, I just avoid all the people who have the habit of sharing this information.*

In summary, each narrative sheds light on the insidious impact of Haiti’s turmoil on individual health outcomes, exposing the harsh reality experienced by its residents. Hypertension, anxiety, depression, sleep disturbances, substance abuse, suicidal thoughts, and post-traumatic stress symptoms manifested as symptoms of a deeper, more pervasive affliction. Throughout the focus groups, participants articulated the pivotal role of familial and peer support, spiritual and religious practices, engagement in music or enjoyable activities, and intentional disengagement from news sources fixated on the country’s current state.

## Discussion

The main goal of this study was to explore the mental well-being of Haitians in Haiti and the United States amidst severe political crises, focusing on individual lived experiences, barriers, and facilitators. Through qualitative analysis of focus groups conducted with 28 Haitian men and women across Haiti and the US, poignant themes emerged, spotlighting the profound impact of Haiti’s deteriorating situation on individual and communal well-being. Participants’ narratives underscore the severe health burden stemming from chronic exposure to socio-political instability, with reports of significant psychological distress and physical health complications such as hypertension, heart conditions, anxiety, substance abuse, suicidal ideations, sleep problems, depression, and PTSD. Our findings align with abundant literature demonstrating that sociopolitical violence, a social determinant of mental health factors, has a significant negative impact on human capital and labor market outcomes, with varying effects on men, women, and children (24). It also leads to higher levels of depression, hostility, and PTSD, particularly in adolescents (25).

Systemic and Environmental Risk Factors and the Urgency of Culturally Attuned Interventions.

We underscore that the prevalent distress in Haitian communities is not just a byproduct of current adversities but reveals a community grappling with the compounded and unending foreign interference in political affairs. Exposure to historical traumas, such as race-based enslavement, decades of US Occupation, Independence ransom from former French colonizers, 30 years of Duvalier dictatorship, colonization, and natural disasters, has had a significant impact on the mental health and resilience of Haitians (26). Following the Haiti 2010 Earthquake, Cénat et al.’s (27) meta-analysis revealed that a significant proportion of the population suffers from severe mental health issues post-trauma. Specifically, around 28% have exhibited severe PTSD symptoms, 32% have reported severe depression, and 20% have experienced severe anxiety. These conditions co-occurred with other mental health concerns such as distress, suicidal thoughts, and increased alcohol consumption. Their data suggested a gender disparity, with females being over 40% more likely to endure severe PTSD symptoms compared to males.

More than 10 years following this devastating natural disaster in Haiti, as the country is undergoing one of the deadliest socio-political disasters in its history, our exploration of Haitian lived experiences with mental health reveals critical health risk factors, including ongoing gang violence, disruption in daily lives, limited access to health services, cultural insensitivity in existing care frameworks, and pervasive stigma against mental health issues. These factors exacerbate the community’s vulnerability, highlighting an urgent need for culturally attuned interventions that address the specificities of the Haitian experience.

In recent years, there has been a growing recognition of the imperative to assess the colonial legacies and efficacy gaps inherent in solely etic, Western-based psychological interventions, leading to an increased emphasis on indigenous healing modalities (28). Haiti has historically pioneered culturally attuned mental health practices, exemplified by initiatives such as the Ligue Nationale d’Hygiène Mentale (27). Furthermore, the concept of resilience, often used to characterize the endurance of the Haitian populace amidst violence and political exploitation, has been scrutinized for its tendency to overlook Haiti’s psychological needs (29).

Our focus group discussions vividly depict the multigenerational distress, stressing the importance of interventions that cater to the diverse needs of all segments of the population, including children, youth, women, and the older adult, who are particularly vulnerable (30). Education and culturally appropriate interventions emerged as pivotal tools in this endeavor, serving to destigmatize mental health issues, foster emotional literacy, and empower communities with knowledge and resources for resilience and intergenerational healing (see Table 3). For Haitians, recognizing and amplifying the cultural fortitude that has withstood the original wounds of colonial subjugation and subsequent traumas is paramount in our healing odyssey. Historical trauma/intergenerational traumas theoretical framework provides a lens to comprehend the link between structural health determinants and the collective oppression experienced by Indigenous/African descent peoples across generations, with an emerging emphasis on healing factors. This approach can be extended to Haitians, centering on intergenerational recovery and well-being (31–34).

**Table 3:** Integrated mental health strategy for Haitian communities: policy recommendations and action plan.

#	Problems identified	Policy recommendation	Indicators	Risk indicators
1	Chronic Traumatic Stress: Gang violence, unemployment, lack of social support, forced displacement	Restoration of security in Haiti. Investment in development programs. Enabling displaced individuals to return home for family reunions. Implement trauma-informed interventions within communities.	Reduction in gang affiliation, homicide, and gender-based violence (GBV) rates. The number of families reunited and relocated. % of economic growth	Political instability and program engagement.
2	Increased Health Burdens: Cardiovascular risks, heart diseases, depression, suicide, sleep problems, PTSD	Invest in health promotion campaigns. Implement universal health coverage to expand access to comprehensive health services.	Campaign implementation and reach. Service accessibility and utilization.	Financial constraints and logistical challenges.
3	Structural Risk Factors: Limited access, cultural responsiveness, hopelessness, opportunity scarcity, stigma	Invest economically in the Haitian public health sector. Implement universal health coverage and reform mental health training to enhance accessibility to mental health services.	Increased service utilization. Accessibility of services.	Cultural barriers and funding limitations.
4	Future Uncertainty	Develop resilience-building programs geared toward the future	Indicators of resilience factors within the community and reduction of multidimensional crisis	Ongoing crises and levels of community participation.
5	Multigenerational Concerns: School closures, family dislocation, children’s and caregivers’ wellbeing	Initiate mental health programs for youth and families through schools, churches, media, and community centers. Launch non-violent education campaigns.	Program uptake and effectiveness; rates of school reopening; decrease in emotional and behavioral symptoms among youth and parents.	Accessibility and sustained funding.
6	Healthy Coping and Protective Factors: Network support system, creative outlets, spiritual practice, and stress management.	Develop and implement programs and media campaigns to teach positive coping skills at the personal, family and local levels (schools, spiritual and religious places, and workplace)	Number of Haitians with access to healthy behavior and coping material dissemination. Reduction in mental health complaint and reported organized crimes.	Lack of awareness and discussion of positive and protective healing factors. Limited means to implement such programs.

There is a need for an exploratory investigation into historical accounts of healing practices among Haitians during the eras of enslavement, US invasion, and dictatorship to provide insight into ancestral resilience and healing practices. In their exploratory analysis, Henderson et al. (35) delved into the historical practices of healing carried out by enslaved African individuals on plantations in the Southern United States. Their study highlighted two primary coping strategies utilized by these healers and those in search of healing: devising coping mechanisms and active resistance. Through an analysis of historical documents, their study brought to light the continuous passage of healing knowledge and practices that have contributed to the resilience and wellness of African American lineages over time.

### Limitations

The study is not without limitations, including potential biases in the qualitative analysis, the representativeness of the sample, and the challenges of virtual focus group engagement. Future research should aim for a more representative sample and refine data collection methodologies to enhance the understanding of mental health within the broader Haitian community.

## Conclusion and future directions

In conclusion, the study’s insights underscore the critical need for restoring security in Haiti, emphasizing the social determinants of health, and advocating for policies (see Table 3) that foster transformative policies and mental health education. The provision of sustainable, culturally sensitive services, public health strategies to address the crisis, and investment in the youth is crucial for the well-being of the Haitian population. Derived from the community’s lived experiences and propositions, these recommendations serve as a blueprint for a holistic approach to mental health and multigenerational healing in Haitian communities.

## Data Availability

The raw data supporting the conclusions of this article will be made available by the authors, upon IRB approval.

## Footnotes

^1^Humanitarian parole program of the Biden-Harris Administration, allowing Haitian individuals to enter the US if they have a sponsor.
